# Evaluating the potential of novel genetic approaches for the treatment of Duchenne muscular dystrophy

**DOI:** 10.1038/s41431-021-00811-2

**Published:** 2021-02-09

**Authors:** Vratko Himič, Kay E. Davies

**Affiliations:** Department of Physiology, Anatomy and Genetics, University of Oxford, Oxford, UK

**Keywords:** Genetics, Diseases

## Abstract

Duchenne muscular dystrophy (DMD) is an X-linked progressive muscle-wasting disorder that is caused by a lack of functional dystrophin, a cytoplasmic protein necessary for the structural integrity of muscle. As variants in the dystrophin gene lead to a disruption of the reading frame, pharmacological treatments have only limited efficacy; there is currently no effective therapy and consequently, a significant unmet clinical need for DMD. Recently, novel genetic approaches have shown real promise in treating DMD, with advancements in the efficacy and tropism of exon skipping and surrogate gene therapy. CRISPR-Cas9 has the potential to be a ‘one-hit’ curative treatment in the coming decade. The current limitations of gene editing, such as off-target effects and immunogenicity, are in fact partly constraints of the delivery method itself, and thus research focus has shifted to improving the viral vector. In order to halt the loss of ambulation, early diagnosis and treatment will be pivotal. In an era where genetic sequencing is increasingly utilised in the clinic, genetic therapies will play a progressively central role in DMD therapy. This review delineates the relative merits of cutting-edge genetic approaches, as well as the challenges that still need to be overcome before they become clinically viable.

## Introduction

Duchenne muscular dystrophy (DMD) is a lethal X-linked recessive disorder that is caused by loss-of-function variants in the dystrophin gene. Dystrophin is a structural protein that tethers muscle to surrounding extracellular matrix [1]. This progressive wasting disease affects around 1 in 5000 boys, who lose ambulation around the age of 12, and require ventilation by the age of 18. Current clinical management options, such as corticosteroid therapy, can only delay the loss of ambulation; there is no effective cure for DMD. Premature death occurs between the second to fourth decade with respiratory complications and dilated cardiomyopathy being the most common causes of death [1, 2].

**Fig. 1:**
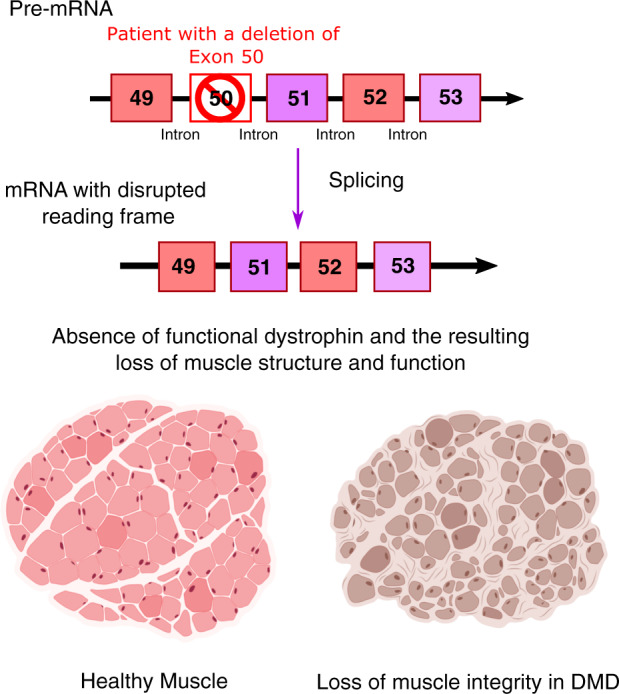
Variants in the DMD gene lead to the production of non-functional dystrophin. In a patient with a deletion in exon 50, the splicing process produces mRNA with a disrupted reading frame, leading to the absence of functional dystrophin.

Since DMD has a genetic cause (Fig. 1), and restoration of the disrupted reading frame is required [1], targeting the affected muscle cells with genetic approaches has curative potential. As the therapeutic developments for DMD are reviewed thoroughly elsewhere [2], the purpose of this review is to specifically evaluate the potential of novel genetic approaches for DMD treatment, a promising subset of the potential therapeutic repertoire. It further considers the biomedical, technical, and regulatory challenges that could preclude these approaches from becoming successful therapies.

## Drug development for DMD is challenging

The development of curative therapies for DMD is challenging for a number of reasons [1]. Firstly, the disease does not discriminate between regions of the body and affects muscle globally—crucially involving the myocardium and the diaphragm. Secondly, muscle is a post-mitotic tissue, and therefore simply halting dystrophin loss will not replace muscle that has already been lost [2]. Additionally, because of the systemic and permanent changes made by some of these novel genetic approaches, the animal models used to evaluate these therapies need to be scrutinised in their similarity to the clinical progression seen in humans [3]. Whilst some humanised mouse strains do exist, validation of these novel genetic methods in larger animal models such as dogs and monkeys is necessary as they resemble the human DMD phenotype more closely [3].

## Current therapy options are lacking

**Fig. 2:**
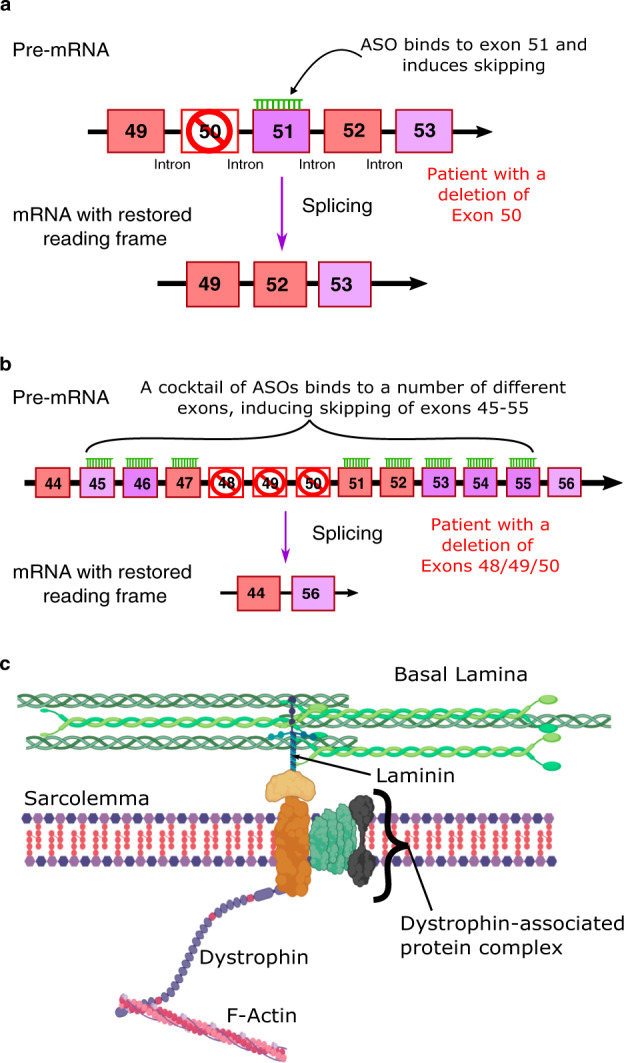
Improvements of multi-exon skipping in comparison to single exon skipping. **a** Single exon skipping is only suitable for patients with a specific variant (exon 50 here), whereas (**b**) multi-exon skipping can help restore the reading frame in patients with different variants (either in exons 48, 49 or 50 in this example) and (**c**) still produce a functional dystrophin protein.

A limited number of therapies targeting the primary genetic defect are available to patients. Exon skipping involves the delivery of antisense oligonucleotides (ASOs) that bind to the pre-mRNA dystrophin transcript and induce skipping of certain exons [4]. This produces a milder phenotype, similar to that seen in Becker muscular dystrophy, a less severe dystrophinopathy. So far, three therapies that induce the skipping of a single exon have received Food and Drug Administration (FDA) approval; eteplirsen (exon 51), golodirsen (exon 53) and most recently accelerated approval for viltolarsen (exon 53). All three of these ASOs utilise the phosphorodiamidate morpholino oligomer (PMO) backbone. However, these approved therapies have their limitations [5]. Single nucleotides cannot enter muscle cells with adequate efficiency, and this has been reflected in the relatively modest increases in protein production. However, due to the lack of effective treatments these single exon skipping therapies are seeing success at the regulatory level; casimersen (exon 45) has recently been accepted for review by the FDA despite a mere 1.74% increase in mean dystrophin production [6]. Additionally, DMD patients have variants in different exons, causing varied reading frame disruptions. Single exon treatments are therefore only applicable to a small subset of the DMD patient population that they were designed to target; eteplirsen, for example, is suitable for around 14% of DMD patients (Fig. 2a) [7]. In 2014, the European Medicines Agency (EMA) approved ataluren, a premature stop codon readthrough therapy for patients with nonsense variant DMD [2]. Neither ataluren, nor the exon skipping therapies have reported beneficial effects on the heart.

## Improving on single exon skipping

Current exon skipping approaches have questionable efficacy and can only treat a small subset of patients with a specific exon variant. New emerging methods can now tackle both of these drawbacks.

Multi-exon skipping could overcome the limited scope of single exon skipping, enabling the treatment of a larger proportion of DMD patients with variants in different exons (Fig. 2b) [8]. It concomitantly allows the choosing of a truncated dystrophin protein which is more stable and functional. Recently, early multi-exon skipping treatment in neonate dogs was demonstrated to be both safe and efficacious. While dystrophin was restored to only 14% of healthy levels, the dogs exhibited significant functional improvements [9]. Furthermore, a cocktail of ASOs targeting the variant hotspot of exons 45–55 efficiently skipped these exons in both immortalised DMD patient muscle cells and humanised mice [10, 11]. If this finding is successfully translated to the clinic, it could potentially treat more than 65% of the DMD patient pool [12]. Despite promising pre-clinical results, there are some regulatory challenges with this approach [8]. Whilst multi-exon skipping treatments have efficacy when delivered as a cocktail of ASOs, individual oligonucleotides do not necessarily have sufficient efficacy on their own. This is an issue as the current regulatory framework states that each individual ASO in the mixture has to be evaluated separately [13].

Muscle-homing peptides could be a way to produce a step-size increase in efficacy of exon skipping treatments. Peptide-conjugated PMOs (PPMOs) have been synthesised by linking muscle-homing peptides to the PMO backbone of ASOs, improving their pharmacokinetic profile [14]. They do so by increasing the cellular uptake of the conjugated molecule and increasing the duration that dystrophin is regenerated after the treatment has ceased. Compared to PMOs, PPMOs can double the exon skipping efficacy in both skeletal and cardiac muscle of *mdx* mice [15]. Whilst unconjugated PMOs have little success in targeting cardiac muscle in larger animals, PPMOs successfully restored dystrophin in the myocardium of dogs [16]. Although PPMOs achieve enhanced potency at lower doses, their concerning toxicology profile will need to be examined and addressed [14]. In terms of clinical assessment, Sarepta (Cambridge, Massachusetts, USA) concluded a phase I/IIa trial of a peptide-conjugated version of eteplirsen but are yet to publish results [17]. Sarepta is also examining five additional PPMOs targeting different exons at the pre-clinical stage.

Varying the chemistry of ASOs could also improve their tissue uptake. Two approaches have been used to optimise ASO chemistry, including Tricyclo-DNA ASOs and stereopure ASOs. Mouse studies suggest that tricyclo-DNA ASOs can cross the blood-brain barrier and successfully target the nervous system [18]. This is an encouraging finding as a subset of DMD patients also have cognitive dysfunction [19]. The stereopure ASO (suvodirsen) has been tested in human patients. Despite promising pre-clinical results and an excellent safety profile in human patients, regrettably, suvodirsen did not significantly increase dystrophin levels [20]. Prior to this, suvodirsen received fast track status from the FDA due to promising pre-clinical results. This recent failure highlights the need to evaluate therapies in large animal models and subsequently carry out limited human trials as soon as possible.

## Genome editing

The CRISPR-Cas9 system uses a guide (g)RNA to instruct a Cas9 nuclease to induce a double-strand break (DSB) at virtually any targeted region of the genome [21]. The DSB is then repaired by non-homologous end joining (NHEJ), inducing additional variants in the forms of insertions and deletions. This can restore the reading frame by deleting an additional exon and mimicking the effect of exon skipping drugs [21]. CRISPR-Cas9 has several advantages over exon skipping therapies: it could negate the need for re-injection (as DNA and not pre-mRNA is targeted), and it has ability to treat patients with duplications in certain exons of the DMD gene.

CRISPR-Cas9 has restored the reading frame by deleting single exons [22] and has improved muscle function in *mdx* mice [23]. Recently, deletion of exon 51 in a pig model lacking exon 52 led to widespread dystrophin expression, including in the heart and diaphragm [24]. This efficacy was replicated in induced pluripotent stem cell (iPSC)-derived cardiomyocytes and myoblasts from a patient with the same exon 52 deletion. In both cases the treatment decreased arrhythmia susceptibility [24]. CRISPR-Cas9 could also be used to induce multiple deletions and mimic multi-exon skipping. The use of a multiplexed gRNA targeting the variant-prone exons 45–55 restored in vitro dystrophin expression in patient-derived myoblasts. When these myoblasts were implanted into mice, expression was maintained [25]. Multi-exon deletion has also been achieved in human iPSCs in exons 3–9 [26] and in exons 52–53 in a mouse model with 34% dystrophin expression in cardiac myofibres [27].

Despite these promising pre-clinical findings, certain challenges could preclude CRISPR-Cas9 from becoming a successful therapy [28]. Even though Cas9 is instructed to induce DSBs by the gRNA in a targeted manner, off-target DNA cutting still remains an issue. This can cause unwanted additional variants in other genes or indeed in other exons in the dystrophin gene. Encouragingly, improved gene editing efficiency has recently been achieved when nuclear localisation signals were added to the Cas9 nuclease construct [29]. In addition, CRIPSR editing can be made more reliable by optimising the gRNA design or using high fidelity Cas9 [30]. Furthermore, the identification of humoral immune responses to gRNAs and pre-existing adaptive immunity to Cas9 homologues has meant that gene editing now also faces an immunological barrier [31]. As infections by *Staphylococcus aureus* and *Streptococcus pyogenes* are relatively common, some patients have high levels of antibodies and T cells against these bacterially-derived nucleases [32]. Interestingly, the age at which patients are eventually treated could tackle this immunogenicity; the treatment of neonatal mice, as opposed to adult mice, avoids the humoral response to Cas9 [28]. Another possibility to mitigate this type I interferon response is to remove the 5’-triphosphate group from gRNAs [33]. Finally, one of the challenges to effective CRISPR therapy is the long-term restoration of function. Encouragingly however, CRISPR editing of 6-week old *mdx* mice led to rescue of both skeletal muscle and also improved cardiac haemodynamics [34].

Despite these limitations, CRIPSR-Cas9 is making large in-roads into effective and safe somatic gene therapy, exemplified by the recent progress with CRISPR-Cas9-edited T cells for cancer immunotherapy [35]. Whilst in this example the gene editing took place ex vivo, a growing number of trials using CRISPR-Cas9 in vivo are actively recruiting. CRISPR-Cas9 has significant potential to cure DMD with a ‘one-hit’ advantage, whereby a permanent alteration of the underlying genome in satellite cells (muscle stem cells) will negate the need for repeated treatment [36].

## RNA editing

RNA editing is a hitherto underappreciated genetic method that could be a viable alternative to CRIPSR-Cas9. When conjugated with a gRNA, this novel approach enables site-directed pre-mRNA editing [37]. Whilst CRIPSR-Cas9 uses the bacteria-derived Cas9 protein, RNA editing utilises human-derived adenosine deaminases acting on RNA (ADARs), bypassing the immune response generated by Cas9. Encouragingly, RNA editing has recently shown promise in treating DMD by restoring dystrophin expression in *mdx* mice [37]. However, relative to CRISPR-Cas9, this technology is still in its infancy, and is not without challenges. ADARs can only make certain chemical alterations and are less efficient than CRIPSR-Cas9. More recently, using SNAP-tagged ADARs has improved both the efficiency and specificity of the RNA editing [38]. Additionally, conjugating ADARs to dCas13 (a catalytically inactive enzyme) has increased the variety of chemical alterations that can be achieved with RNA editing [39]. However, whilst improving efficiency, efforts to improve RNA editing in this way may lead to the aforementioned undesired effects of CRISPR-Cas9. Whilst less powerful than CRISPR-Cas9, RNA editing could be, with further development, a credible approach to minimise pathology progression.

## Surrogate gene therapy and targeting disease mechanisms

Initial efforts to treat DMD involved delivering full-length dystrophin to muscle tissue. However, dystrophin is too large to fit into an adeno-associated viral (AAV) vector, and whilst lentivirus can deliver dystrophin in its full form and allow genome integration (prolonging expression in tissues) [40], the high titres required for it to target muscle are still a challenge. Attention has consequently turned to surrogate gene therapy, where dystrophin alternatives can be delivered to tissue and restore partial functionality [41]. Long-term therapy with micro-dystrophin (μDys), a truncated version of dystrophin, has been shown effective in a canine model [42]. Whilst the persistence of this transgene in tissue is not well known, initial results from an ongoing clinical trial with μDys show that over 80% of the muscle fibres were micro-dystrophin positive with significant expression of μDys in post-treatment biopsies (95.8% compared to normal) [43]. However, μDys may not contain all of the functional elements of full-length dystrophin, particularly its mechanical and scaffolding roles that are important in transmitting forces through interactions with other proteins. In response to this, novel variants of μDys with improved performance, created by modifying the central rod domains, have been developed [44] and are currently in clinical trials.

**Fig. 3:**
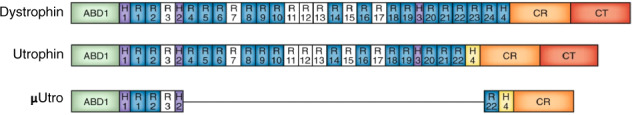
The principles of surrogate gene therapy, with μUtro as an example. Surrogate microgene therapy can improve muscle function and structure. In comparison to dystrophin, μUtro also encodes the actin-binding domain (ABD1). This microgene is effective despite lacking the C-terminal (CT) domain and having only three hinge (H) domains, four spectrin-like repeats (R) and the cysteine-rich (CR) domain. (Adapted from Davies and Chamberlain [41] with permission).

Recently, miniaturised utrophin (μUtro), a shortened codon-optimised version of utrophin (that differs in some protein–protein interactions from, and is itself a surrogate of, dystrophin), effectively treated DMD in *mdx* mice [45]. Crucially, it prevented muscle pathology and was non-immunogenic in large dog models (Fig. 3) [45]. Whilst muscle deterioration was halted, due to the juvenile (and not neonatal) age at which the dogs were treated, a potential reversal of the phenotype was, however, not confirmed.

A third option is overexpressing GALGT2, which stimulates the upregulation of key cytoskeletal binding proteins that can act as surrogates of dystrophin. *GALGT2* therapy can prevent ventricular remodelling and fibrosis in the hearts of *mdx* mice [46] and has also been successful in larger animal models like the rhesus macaque [47]. After demonstrating safety in pre-clinical models, this therapy is now being evaluated in a phase I/IIa trial to evaluate its safety in humans, with results expected towards the end of 2020 [48].

Directly targeting disease mechanisms with genetic approaches is another possibility. The cytosolic calcium overload that occurs in tissues can be reduced with AAV-mediated sarcolipin silencing [49] or increased SERCA2a expression in tissues [50].

## Challenges of delivery

At present, therapies are almost exclusively delivered with viral vectors [51]. The main challenge is to achieve sufficient dissemination throughout all muscle tissue without adverse effects. The key concerns of high-dose AAV treatment are liver toxicity and the dose-dependent innate immune response generated against the vector [52]. It is now possible to decrease the dose of the vector to mitigate these limitations; the use of a self-complementary AAV to deliver gRNA achieves sufficient CRISPR-Cas9 editing efficiency with a 20-fold lower dose than with the previous single-stranded AAV [53].

The vector itself is a limiting step for many of these novel genetic approaches; there has to be a revolution in the efficiency, tropism and yield of viral vector production in order for the therapies to become commercially and clinically viable [51]. When delivered with a viral vector, CRISPR-Cas9 activity may be prolonged longer than necessary, compounding the likelihood of off-target editing and the mounting of an immune response [28]. Recently, alternatives to AAV have been explored and show promise in improving CRISPR-Cas9 therapy. Excitingly, the use of extracellular nanovesicles to deliver gene editing machinery achieved permanent exon skipping in *mdx* mice and achieved 90% exon skipping in iPSC-derived muscle cells from DMD patients [54]. Furthermore, gold nanoparticles have the capacity to deliver Cas9 as well as donor DNA; this would enable cells to repair the Cas9-induced DSB with homology directed repair [55]. This is more challenging than deleting an exon (as is done with NHEJ), but if successful, could restore the expression of the wild-type dystrophin gene.

## Challenges of delivery to the clinic

In addition to challenges with delivery to muscle tissue, hurdles still remain in translating these therapies to the clinic. Firstly, well-designed clinical trials are essential. The aforementioned eteplirsen was rejected twice by the EMA [56], and its approval by the FDA was highly controversial with high patient advocacy at the regulatory level. The choice of primary outcome measures in the evaluation of these novel approaches will be crucial. The six-minute walk test (6MWT) was the primary outcome measure for the eteplirsen trial and its use is almost ubiquitous in previous studies. The impacts of motivation, learning and pressure on patients by family members can make this an unreliable measure [56]. The assumption that one can achieve significant functional benefit without an increase in cytoskeletal protein production is a blind and dangerous assumption stemming from the pervasiveness of the 6MWT [57]. Although the more comprehensive North Start Ambulatory Assessment (NSAA) is now being used in the majority of trials [57], more objective measures are needed in order to guarantee safety and efficacy for both patient and family in this serious and devastating disease. Encouragingly, the limited data from a small number of patients treated with these novel genetic approaches seems transformative. We could therefore become less dependent on endpoints such as the 6MWT and the NSAA, as measuring increases in protein level following gene therapy can give a more clear and reliable evaluation of efficacy. Recent advances make this effort more achievable—for example, progress in the standardisation of dystrophin quantification in muscle biopsies [58] as well as the development of non-invasive biomarkers to monitor the tissue responses to both disease progression and intervention [59].

Additionally, the success of these novel approaches will be closely dependent on early detection and treatment. Country-wide neonatal screens for DMD are needed, as early therapy could halt the loss of dystrophin before significant functional deterioration takes place [60]. However, ‘early’ does not necessarily mean at the earliest opportunity; premature gene therapy or exon skipping could mean that the muscles are still growing and there may be a loss of benefit in muscle synthesised post-treatment. CRISPR-Cas9 overcomes this challenge as it has an advantage: it alters the DNA itself and so treatment could be given at the earliest opportunity. For those novel genetic approaches that eventually progress through clinical trials, they will be most effective as combinatorial therapies [61]. Corticosteroids are needed to dampen the immunogenic effect of vectors, bacteria-derived enzymes or muscle-homing peptides. Encouragingly, with certain therapies such as exon skipping, the immunosuppressive drugs can be withdrawn following functional restoration of dystrophin expression. Additionally, as surrogate gene therapy is unlikely to completely restore function, combining different genetic approaches could be a potential treatment option. Interestingly, the combination of overexpression of utrophin and increased dystrophin expression through exon skipping confers additive functional benefits [62].

Lastly, given the variability in disease progression between DMD patients, a more personalised approach to therapy will be needed [63]. DMD patients with a more rapid and severe clinical progression can now be identified, such as those with variants in the Tctex1 domain containing 1 (*TCTEX1D1*) gene which is associated with cardiomyopathy severity [64]. These, among other variants, could be used as both prognostic markers and potential novel therapy targets.

## Concluding remarks

Novel genetic approaches show significant potential in transforming treatment for DMD. In light of the progress being made in somatic gene editing, CRIPSR-Cas9 therapy can, within the next decade, become an effective cure for DMD. Large strides are being made in improving the delivery and safety of CRISPR-Cas9—the two main bottlenecks of this technology. Early and ideally pre-symptomatic diagnosis of DMD will allow treatment at the ideal point in the clinical progression of the disease. This will negate the need for re-injection and prevent ambulatory loss that would have otherwise occurred. The treatment of DMD with genetic approaches is as much of an immunological challenge as it is a genetic one. It is therefore clear that, whilst these novel genetic approaches are impressive and show serious potential, a robust treatment plan will require a combinatorial approach with multidisciplinary care as opposed to a one-size-fits-all strategy. Whilst blockbuster new treatments are desirable, a clear aim should be to restore sufficient function to patients. A patient-focused approach to drug development and clinical trial design is important with any disease, and this patient centricity is particularly crucial in rare genetic diseases such as DMD, where conventional drug development thresholds may not be so suitable.
